# Does Diacylglycerol Accumulation in Fatty Liver Disease Cause Hepatic Insulin Resistance?

**DOI:** 10.1155/2015/104132

**Published:** 2015-07-26

**Authors:** Brian N. Finck, Angela M. Hall

**Affiliations:** Washington University School of Medicine, St. Louis, MO 63110, USA

## Abstract

Numerous studies conducted on obese humans and various rodent models of obesity have identified a correlation between hepatic lipid content and the development of insulin resistance in liver and other tissues. Despite a large body of the literature on this topic, the cause and effect relationship between hepatic steatosis and insulin resistance remains controversial. If, as many believe, lipid aggregation in liver drives insulin resistance and other metabolic abnormalities, there are significant unanswered questions as to which lipid mediators are causative in this cascade. Several published papers have now correlated levels of diacylglycerol (DAG), the penultimate intermediate in triglyceride synthesis, with development of insulin resistance and have postulated that this occurs via activation of protein kinase C signaling. Although many studies have confirmed this relationship, many others have reported a disconnect between DAG content and insulin resistance. It has been postulated that differences in methods for DAG measurement, DAG compartmentalization within the cell, or fatty acid composition of the DAG may explain these discrepancies. The purpose of this review is to compare and contrast some of the relevant findings in this area and to discuss a number of unanswered questions regarding the relationship between DAG and insulin resistance.

## 1. Introduction

Hepatic insulin resistance, lipid accumulation, and inflammation seem to be tightly interconnected. Indeed, strong correlations among these variables have been detected in obese human subjects and in studies conducted on a variety of mouse and rat models. However, the cause and effect relationship among these factors is not always clear and usually difficult to discern [1]. Furthermore, the largest genetic predictors of NAFLD are not always associated with hepatic insulin resistance [2, 3]. Experimental approaches to modulate the abundance of a given lipid unavoidably lead to changes in the abundance of other interconverted lipids such that manipulating the concentrations of one lipid in isolation seems impossible. Furthermore, difficulties in measuring the abundance of lipids present at very low levels and reproducibility across different model systems have raised questions regarding whether a specific lipid can be causally linked to development of insulin resistance. Whereas several lipids accumulate in steatotic liver, this review will focus on diacylglycerol (DAG), the evidence linking it to insulin resistance, and the controversy surrounding this linkage.

On the surface, DAG is a simple hydrophobic lipid that is normally a component of cellular membranes or is stored in lipid droplets. DAG is composed of a glycerol backbone and two fatty acyl groups. However, the biophysical properties and the physiological effects of DAG can be strongly influenced by the composition of the fatty acyl groups and its physical location within the cell. For example, acyl moieties can be esterified at either the sn-1,2 or the sn-1,3 positions of glycerol depending upon the pathway used to generate the DAG molecule. These two stereoisomers have different biophysical properties in membranes and previous work has shown that the sn-1,2 stereoisomer is much more potent, compared to sn-1,3-DAG, at activating certain signaling cascades linked to insulin resistance [4]. Accumulation of DAG containing saturated fatty acids has also been linked to development of insulin resistance. There is also correlative evidence that the abundance of DAG in the various intracellular compartments (membrane versus lipid droplet) can be more strongly associated with insulin resistance [5, 6].

## 2. Connections between DAG and Insulin Resistance

Some of the original work correlating tissue DAG concentrations to insulin resistance was conducted in obese rats almost 25 years ago [7]. Based on previous work showing that phorbol ester, a DAG analog, could impair insulin action, Turinsky and colleagues hypothesized that endogenous DAG might be increased in insulin-resistant rodents. Measurement of DAG in obese Zucker rat tissues revealed that 1,2-DAG was elevated in multiple tissues in this model of type 2 diabetes. Since then, elevated DAG has been correlated to impaired insulin action in a variety of studies [8–13]. Ectopic accumulation of DAG in liver may be due to a variety of factors including consumption of a high fat or high sugar diet, inability of adipose tissue to appropriately store lipids leading to elevated circulating free fatty acids [14–16], or effects of oxidative stress in the liver causing DAG formation [17–19]. Because many lipids accumulate ectopically in obesity, which is correlated with insulin resistance, a variety of lipid species can be correlated with insulin resistance in obese animal models or humans. However, much of the recent attention in this area has focused on DAG due to the identification of clear mechanisms linking DAG to impaired insulin signaling.

**Figure 1:**
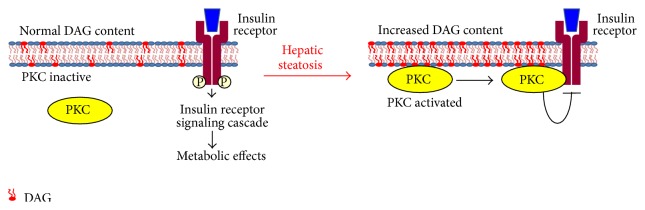
Proposed mechanism for DAG-mediated insulin resistance through activation of PKC is shown.

Specifically, DAG has been shown in a variety of model systems to activate protein kinase C (PKC) family kinases, which physically interact with membrane-embedded DAG [20]. In hepatocytes or intact liver, links among DAG accumulation, PKC activation, and impaired insulin action have been made for PKC*ε* [21] and PKC*δ* [22]. The mechanism of PKC*ε* inhibition of insulin action was mediated via a direct interaction of PKC*ε* with the insulin receptor to inhibit its intrinsic kinase activity [21] (Figure 1). The link between PKC*ε* activation and hepatic insulin resistance is supported in correlative fashion by several papers [12, 13, 16, 23, 24] and in more convincing fashion by “knocking down” PKC*ε* in liver by RNAi [21]. PKC*ε* knockout mice exhibit improved glycemic control on a high fat diet, but this is likely mediated via enhanced insulin secretion [25]. PKC*δ* is also activated in steatotic liver [22, 25] and PKC*δ* knockout mice are protected from high fat diet induced hepatic steatosis while PKC*δ* overexpression was sufficient to drive insulin resistance [22].

As delineated above, numerous studies have correlated altered DAG concentrations to PKC activation and insulin resistance. However, it should be noted that several notable exceptions to these correlations have been detected where DAG accumulation in liver was not associated with development of insulin resistance [26–33]. One interpretation of these data is that DAG elevation is not,* per se*, sufficient to cause insulin resistance. The caveat to this conclusion is that it is not always clear whether all species, stereoisomers, or subcellular compartments are affected similarly. Could a change in the ratio or absolute amounts of sn-1,2 and sn-1,3 affect downstream signaling cascade activity? Similarly, could the chain length and degree of saturation also impact interpretation of these findings? Lastly, the subcellular compartmentalization of DAG has been reported to impact whether DAG accumulation drives insulin resistance or not [5, 6].

Though a large number of studies have examined the correlation between DAG and insulin resistance, this review is going to focus primarily on data generated by targeting enzymes that directly synthesize or degrade DAG. These studies were mostly conducted in animal models with gene deletion, gene expression knockdown, or overexpression. As discussed below, there are multiple enzymatic reactions involving glycerolipid and phosphoglycerolipid substrates that can result in DAG synthesis. The compartmentalization of many of these pathways, the substrate used, and the subcellular location where the reaction occurs could influence the resulting effect on metabolism and signaling. Moreover, despite the focus on enzymes that directly regulate DAG synthesis or turnover, this is not to say that other lipids derived from DAG or substrates for DAG synthesis are not affected. Indeed, as noted above, it is practically impossible to affect the concentration of one lipid in isolation.

## 3. Mechanisms for DAG Synthesis

**Figure 2:**
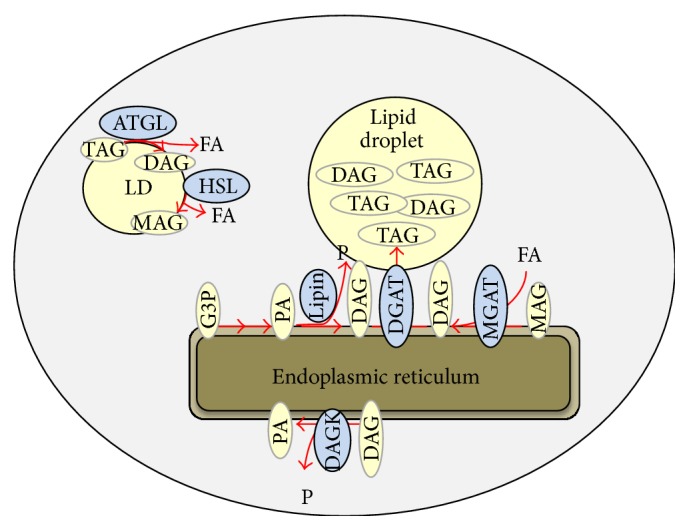
The pathways for DAG synthesis and hydrolysis are shown. FA: fatty acid, P: phosphate, G-3-P: glycerol-3-phosphate, PA: phosphatidic acid (PA), MAG: monoacylglycerol, MGAT: MAG acyltransferase, DAG: diacylglycerol, DGAT: DAG acyltransferase, TAG: triacylglycerol, ATGL: adipose tissue triglyceride lipase, HSL: hormone sensitive lipase, and DAGK: DAG kinase.

In the liver, one of the primary pathways for synthesizing DAG is from the dephosphorylation of ER membrane-embedded phosphatidic acid (PA) by the lipin family of proteins (lipin 1, lipin 2, and lipin 3) (Figure 2) [34, 35]. This pathway can only produce 1,2-DAG since PA is phosphorylated at the sn-3 position. Evidence exists that both lipin 1 and lipin 2 encode significant hepatic PAP activity [36] and may play a role in development of NAFLD and related metabolic abnormalities. Acute adenoviral-mediated knockdown of lipin 1 or lipin 2 was shown to reduce hepatic DAG, PKC*ε* activation, and associated insulin resistance [37, 38]. However, when hepatic steatosis was examined in liver-specific lipin 1 knockout mice fed a diet containing high amounts of ethanol, alcoholic hepatic steatosis and liver diseases were exacerbated by lipin 1 deficiency [39]. Similarly, we have recently found that liver-specific lipin 1 knockout mice are not protected from hepatic steatosis and insulin resistance after high fat diet (our unpublished results). It is not clear whether these discordant results between knockout mouse studies and RNAi approaches are due to duration of lipin inhibition, chronic compensatory mechanisms, or some other experimental differences.

Monoacylglycerol acyltransferase enzymes (MGAT1, MGAT2, and MGAT3) also generate DAG by acylating monoacylglycerol (Figure 1), and both sn-1,2 and sn-1,3 DAG can be synthesized by MGAT enzymes. Recent work has suggested that the expression of genes encoding MGATs (Mogats) is markedly induced in human patients with NAFLD [40] as well as rodent models of obesity [29, 41]. Mogat1 knockdown or Mogat2 KO in mice led to a reversal or prevention of insulin resistance in high fat diet fed mice [29, 41–43]. Interestingly, Mogat1 knockdown in diet-induced obese mice, which caused a marked insulin sensitization, did not affect hepatic DAG content or compartmentalization [29, 42]. Despite this, membrane-associated PKC activity was reduced by Mogat1 knockdown, with the caveat that the PKC activity was not increased by the high fat diet compared to low fat controls [29].

Adipose tissue triglyceride lipase (ATGL) is a major hepatic triglyceride lipase [44]. Genetic deficiency in ATGL leads to ectopic lipid accumulation, due to the inability to mobilize stored triglycerides, in a number of tissues including the liver [28, 31]. ATGL deficiency led to hepatic steatosis, but this was not associated with development of hepatic insulin resistance, inflammation, or fibrosis [28, 31, 32, 45], despite the accumulation of DAG [31]. ATGL activity is also controlled by an enhancer protein called CGI-58 [46]. Knockdown of CGI-58 also resulted in accumulation of hepatic lipids, including DAG, but this did not cause insulin resistance in high fat diet fed mice [26]. A follow-up study concluded that loss of CGI-58 caused accumulation of DAG specifically in lipid droplets rather than ectopically in cell membranes, which prevented activation of PKC*ε* signaling [6]. However, this contradicted another previous study by the same group showing a strong correlation between lipid droplet DAG content and insulin resistance in human liver [5]. These contradictory findings have not been reconciled.

## 4. Mechanisms for DAG Degradation

There are multiple enzymes that convert DAG to other chemical forms. This can be accomplished by addition or removal of a fatty acyl molecule or addition of a phosphate group. The effects of some of these pathways have now been examined by using transgenic mouse systems or RNAi methodology.

The terminal step in triglyceride synthesizes the diacylglycerol acyltransferases (DGAT1 and DGAT2). DGATs are well expressed in liver and have been targeted for gene deletion or knockdown by a number of studies. DGAT1 inhibition did not affect insulin sensitivity in high fat diet fed rats, while DGAT2 knockdown reduced hepatic lipid accumulation and improved hepatic and whole body insulin sensitivity [47]. The improvement in insulin sensitivity was correlated with a reduction in hepatic content of DAG and a corresponding reduction in PKC*ε* activity [47]. Liver-specific overexpression of DGAT2 in transgenic mice somewhat surprisingly led to an accumulation of DAG and TAG but, interestingly, did not affect insulin sensitivity [27]. Subsequent analyses of these mice contradicted this and suggested that hepatic insulin sensitivity was impaired [11]. The discrepant results between the two studies have not yet been explained. It is also unclear why DGAT deficiency and overexpression had paradoxical effects on DAG content, though an unexpected increase in DAG was also observed with Mogat1 inhibition [29].

Hydrolysis of a fatty acyl group from DAG by fatty acid lipases is another way to degrade DAG to other chemical forms. Two genes encoding DAG lipases (Dagla and Daglb) have been cloned, but their role in the liver and in hepatic lipid homeostasis seems to be unknown. Hormone sensitive lipase (HSL) was once considered the primary triglyceride hydrolase but is now considered to be primarily a DAG lipase. HSL deficient mice exhibit increased hepatic insulin sensitivity with reduced hepatic triglyceride content [30, 48], while adenoviral-mediated overexpression of HSL also reduced hepatic steatosis [49]. It is not clear whether hepatic DAG content was affected by HSL loss or gain of function, and thus the evidence provided by these studies may not inform us about the linkage between DAG and insulin resistance.

DAG phosphorylation by DAG kinase to produce PA is another mechanism by which DAG concentrations could be affected. Whereas DAG kinase *δ* (DAGK*δ*) activity in skeletal muscle has been linked to obesity-related insulin resistance, no effect of diminished DAGK*δ* activity in liver was detected [50]. Could this mean that DAGK*δ* is not well expressed in liver or that another isoform of this family, of which there are many, could be the predominant form in liver? This has not been explored to our knowledge and it is not clear which, if any, DAGK family members are highly expressed in liver. Future studies may address this question.

## 5. Conclusions

The review of the relevant literature focused on enzymes that directly synthesize or metabolize DAG reveals a pattern of findings that is extremely mixed. Whereas some of the studies support a link between altered DAG content and insulin resistance through PKCs, other works fail to find a relationship. Again, there is an important limitation to interpreting data from this area; all of the generated data are essentially correlative. It is also unclear how the proposed mechanism for PKC*ε*-mediated impairment in insulin action through inhibiting insulin receptor phosphorylation fits with the concept of selective insulin resistance [51]. Selective insulin resistance refers to the observation that although insulin-mediated suppression of gluconeogenic pathways is impaired in insulin-resistant liver, another pathway that stimulates de novo lipogenesis through the sterol response element binding protein (SREBP1) remains intact [51–54]. Though there are somewhat contradictory findings regarding at which step in the bifurcating insulin signaling cascades the selective insulin resistance occurs [52–54], it is generally believed to be downstream of the insulin receptor. Therefore, it is unclear how the PKC*ε*-mediated impingement on insulin receptor activity fits with this widely observed concept and meshes into the broader model of hepatic insulin resistance. Future work will be needed to address these discrepancies and reconcile existing inconsistencies.
